# Melanogenic effect of dersimelagon (MT‐7117), a novel oral melanocortin 1 receptor agonist

**DOI:** 10.1002/ski2.78

**Published:** 2021-11-29

**Authors:** T. Suzuki, Y. Kawano, A. Matsumoto, M. Kondo, K. Funayama, S. Tanemura, M. Miyashiro, A. Nishi, K. Yamada, M. Tsuda, A. Sato, K. Morokuma, Y. Yamamoto

**Affiliations:** ^1^ Sohyaku Innovative Research Division Mitsubishi Tanabe Pharma Corporation Yokohama Japan

## Abstract

**Background:**

The activation of melanocortin 1 receptor (MC1R) on melanocytes stimulates the production of eumelanin. A tridecapeptide α melanocyte‐stimulating hormone (αMSH) is known to induce skin pigmentation.

**Objectives:**

We characterised the properties of a novel oral MC1R agonist dersimelagon (MT‐7117) with respect to its specific binding to MC1R, downstream signalling and eumelanin production in experimental models.

**Methods:**

The competitive binding and production of intracellular cyclic adenosine 3′, 5′‐monophosphate in cells expressing recombinant melanocortin receptors were examined. A mouse melanoma cell line B16F1 was used for the evaluation of in vitro melanin production. The in vitro activity of MT‐7117 was determined with αMSH and [Nle^4^, D‐Phe^7^]‐αMSH (NDP‐αMSH) as reference comparators. The change of coat colour and skin pigmentation were evaluated after repeat administration of MT‐7117 by oral gavage to C57BL/6J‐A^y^/+ mice and cynomolgus monkeys, respectively.

**Results:**

MT‐7117 showed the highest affinity for human MC1R compared to the other melanocortin receptors evaluated and agonistic activity for human, cynomolgus monkey and mouse MC1R, with EC_50_ values in the nanomolar range. In B16F1 cells, MT‐7117 increased melanin production in a concentration‐dependent manner. In vivo, MT‐7117 (≥0.3 mg/kg/day p.o.) significantly induced coat colour darkening in mice. MT‐7117 (≥1 mg/kg/day p.o.) induced significant skin pigmentation in monkeys and complete reversibility was observed after cessation of its administration.

**Conclusions:**

MT‐7117 is a novel oral MC1R agonist that induces melanogenesis in vitro and in vivo, suggesting its potential application for the prevention of phototoxic reactions in patients with photodermatoses, such as erythropoietic protoporphyria and X‐linked protoporphyria.

## Graphical abstract

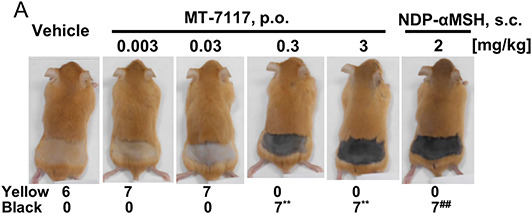

**What is already known about this topic?**

- Activation of melanocortin 1 receptor (MC1R) by agonistic peptide α melanocyte‐stimulating hormone increases eumelanin synthesis in melanocytes.
- Since the common cutaneous manifestation of both erythropoietic protoporphyria (EPP) and X‐linked protoporphyria (XLP) is painful photosensitivity, treatment is based on sunlight avoidance.

**What does this study add?**

- We generated dersimelagon (MT‐7117), a novel orally available and selective agonist for MC1R.
- MT‐7117 increased skin pigmentation in monkeys and darkened coat colour of C57BL/6J‐Ay/+ mice.

**What is the translational message?**

- Increased pigmentation by MT‐7117 may protect patients with photodermatoses, such as EPP and XLP, from phototoxic reactions.
- An oral agent would be a convenient new option for patients with such photodermatoses.

## 1 INTRODUCTION

The melanocortin receptor (MCR) family belongs to the class A family of G‐protein‐coupled receptors and consists of five members: MC1R, MC2R, MC3R, MC4R and MC5R with different tissue distribution and functions.^1^ Expression of MC1R has been reported on various cell types including melanocytes, keratinocytes, monocytes, endothelial cells and fibroblasts.^2^ The activation of MC1R on cutaneous melanocytes by an endogenous ligand, α melanocyte‐stimulating hormone (αMSH), after solar irradiation results in a switch from red‐yellow pheomelanin to brown‐black eumelanin pigment synthesis.^3^, ^4^ On the other hand, αMSH is known to exhibit anti‐inflammatory effects in inflammatory cells.^2^, ^5^ A number of naturally occurring single‐nucleotide polymorphisms of the *MC1R* gene have been identified globally,^6^, ^7^ and variants such as R151C, R160W and D294H in humans are associated with red hair colour and pale skin,^8^ indicating that functional MC1R is crucial in eumelanin synthesis. The major biological functions of eumelanin are absorption of sunlight (photoprotection) and scavenging of free radicals (chemoprotection).^9^, ^10^ Thus, MC1R plays a critical role in the ability of melanocytes to provide an anti‐oxidative defence mechanism against cell damage from harmful solar irradiation.^1^, ^11^

Following activation with agonistic ligands, the Gα_s_ protein dissociates from MC1R and stimulates adenylyl cyclase leading to the intracellular accumulation of the second messenger cyclic adenosine 3′,5′‐monophosphate (cAMP).^2^ Consequently, cAMP activates protein kinase A, which further induces phosphorylation of the transcription factor, cAMP response element‐binding protein and upregulation of transcription factors related to regulation of melanin biosynthetic enzymes including tyrosinase (TYR), tyrosinase‐related protein 1 (TRP1) and dopachrome tautomerase (DCT).^3^, ^12^

The synthetic peptide [Nle^4^, D‐Phe^7^]‐αMSH (NDP‐αMSH), also known as afamelanotide, binds predominantly to MC1R and induces skin pigmentation that prevents phototoxic reactions and it has been approved in several countries for the treatment of adult patients with erythropoietic protoporphyria (EPP). EPP is a lifelong disorder, and photosensitivity that affects EPP patients has a significant impact on their quality of life. However, NDP‐αMSH is not a selective MC1R agonist and thus this may lead to concerns about potential adverse effects, such as nausea, which may be due to binding to MC3R.^13^, ^14^ It is a peptide and thus unsuitable for oral administration due to liability to degradation; it has the limitation of a short half‐life and it needs to be implanted subcutaneously by a healthcare professional every two months. Therefore, there is a need for a selective, safe and orally available MC1R agonist.

We have designed MT‐7117 (dersimelagon phosphoric acid), a novel oral MC1R agonist that is currently in development as a photoprotective agent for patients with EPP and X‐linked protoporphyria (XLP). In this study, we describe the biological profile of MT‐7117 and show its agonistic activity for MC1R in cellular assays, and pigmentation effects in mice and monkeys.

## 2 MATERIALS AND METHODS

### 2.1 Test substances

**FIGURE 1:**
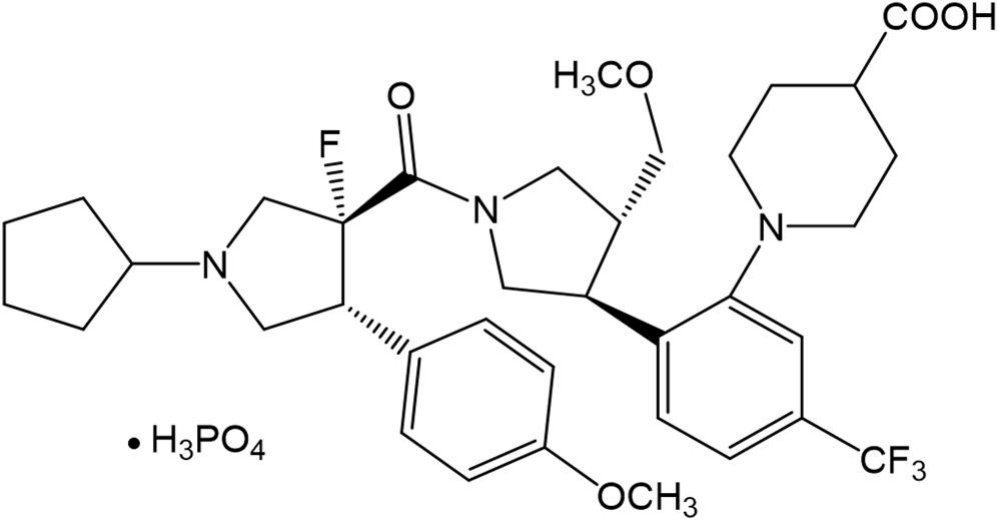
Chemical structure of MT‐7117

MT‐7117 (Figure 1) synthesised at our institute was dissolved in dimethylsulphoxide for in vitro assays or suspended in 0.5% methylcellulose solution or 0.5% methylcellulose plus 0.1% Tween 80 aqueous solution, pH 2.0, for studies in mice and monkeys, respectively. The synthetic method of MT‐7117 is reported separately (manuscript in preparation). αMSH was purchased from PEPTIDE INSTITUTE, INC. NDP‐αMSH was synthesised at PH Japan Co., Ltd.

### 2.2 Cell lines

Human embryonic kidney 293 (HEK293) cells stably expressing melanocortin receptors were established by our institute, including expression of mouse MC1R (mMC1R), rat MC1R, cynomolgus monkey MC1R (cmMC1R), human MC1R (hMC1R) wild type, hMC1R V60L, hMC1R V92M, hMC1R R151C, hMC1R R163Q and human MC4R (hMC4R). The site‐specific integration of each MCR gene was carried out in accordance with the Jump‐In^TM^ TI^TM^ Gateway® Targeted Integration System user guide (www.lifetechnologies.com) except replacement of tetracycline‐induced CMV promoter into SV40 promoter in MCR expression vector as previously described.^15^ This technology enables stable single copy integration at the same location in the genome, equalising expression levels. CHO‐K1 cells expressing human MC2R and accessory protein MRAP were prepared by EuroscreenFast (Gosselies, Belgium). The B16F1 mouse melanoma cell line was purchased from the American Type Culture Collection.

### 2.3 Cyclic AMP assays

Hanks' balanced salt solution containing 1% bovine serum albumin and 10 mmol/l HEPES was prepared and used as an assay buffer. Cells were suspended in the assay buffer containing 0.5 mmol/l 3‐isobutyl‐1‐methylxanthine (Calbiochem) and plated into a microplate. Serially diluted test substances were then added and incubated for 30 min at 37°C. Concentrations of cAMP in the cells were quantified using the cAMP dynamic 2 kit (Cisbio Bioassays) by measuring fluorescence. The maximum responses (*E*_max_) and EC_50_ values (50% effective concentration of the Emax value of αMSH) were calculated.

### 2.4 Radioligand binding assays

Determination of binding affinities for human recombinant MCRs (MC1R, MC3R, MC4R and MC5R) were performed by using [^125^I] NDP‐αMSH radioligand binding. The material of receptors, MC1R (Cat.# ES‐195‐M), MC3R (Cat.# RBXMC3M), MC4R (Cat.# RBHMC4M) and MC5R (Cat#. RBXMC5M) were purchased from PerkinElmer. In brief, test substances and [^125^I] NDP‐αMSH were mixed with human recombinant MCRs in incubation buffer containing 25 mmol/l HEPES, pH 7.0, 100 mmol/l NaCl, 1 mmol/l 1,10‐phenanthroline, 1.5 mmol/l CaCl_2_, 1 mmol/l MgSO_4_ and protease inhibitors for 60 or 120 min at 37°C. After separation of bound and free ligand with washing, radioactivity was measured by a scintillation counter (PerkinElmer, TopCount NXT^TM^). The IC_50_ values were determined with non‐linear regression analysis. The Ki values were calculated using the following formula:

$$\text{Ki}=IC_{50}/(1+[\text{concentration of radioligand}]\;/\;\text{Kd})$$

Kd is the dissociation constant calculated from historical value on assay validity.

### 2.5 Melanin production in B16F1 cell line

Dulbecco's modified Eagle medium without phenol red (Life Technologies), containing 10% foetal bovine serum (Gibco) and 100 U/ml of penicillin‐streptomycin (Gibco), was used as the assay medium. Cells (1.5 × 10^4^) were seeded into a 96‐well culture plate. Serially diluted test substances were added to the plate and incubated at 37°C in a humidified 5% CO_2_ incubator for 3 days. The melanin concentration in the supernatant was determined from the optical density (405 nm) measured by VersaMax^TM^ microplate reader (Molecular Devises) using a standard curve of eumelanin (Sigma‐Aldrich, M8631). The EC_50_ values (50% effective concentration of the Emax value of NDP‐αMSH) were calculated.

### 2.6 Coat colour darkening in Ay/a mice

Unless noted otherwise, 3‐week‐old male C57BL/6J‐A^y^/+ mice (Ay/a mice) were purchased from Japan SLC, Inc. Mice were housed under specific pathogen‐free conditions, and all animal experiments were conducted according to the Guidelines for Animal Experimentation at our institute. On day 0, the coat of the lower back was shaved, and animals were allocated to experimental groups. MT‐7117 was administered orally or NDP‐αMSH was subcutaneously injected once daily for 6 consecutive days. Assessment of coat colour of the shaved area on day 6 was performed macroscopically in a blinded manner. The dorsal skins were dissected and fixed in a neutralised formalin then embedded in paraffin. Melanin production in the hair roots of the dorsal skin was histologically evaluated by Fontana‐Masson staining. For gene expression analysis, pinnae were dissected after single dosage of MT‐7117 to 7‐week‐old male Ay/a mice.

### 2.7 Quantitative real‐time PCR

Total RNA was extracted using the Qiagen RNeasy Mini Kit (Qiagen), and quantitative real‐time PCR for *Tyr*, *Trp1* and *Dct* was performed using the One Step SYBR PrimeScript PLUS RT‐PCR Kit (Takara Bio). Hypoxanthine phosphoribosyltransferase 1 (*Hprt*) was used as an endogenous control for normalisation. The ΔCT method was used to determine the relative expression levels. All PCR primers were purchased from Takara Bio with following product codes, MA153806 (*Tyr*), MA129755 (*Trp1*), MA118342 (*Dct*) and MA031262 (*Hprt*).

### 2.8 Pigmentation on the facial skin of cynomolgus monkeys

This study was conducted in compliance with the ‘Act on Welfare and Management of Animals’ in Japan and the ‘Guidance for Animal Care and Use’ and in accordance with the protocol reviewed by the Institutional Animal Care and Use Committee of the test facility, which is fully accredited by AAALAC International. Female cynomolgus monkeys (*Macaca fascicularis*) aged 2–3 years were assigned to experimental groups to ensure homogenous distribution of the skin colour (L* value) at pre‐test among the groups. The measurement site (cheek) was the same site on the face for all animals. MT‐7117 was administered by oral gavage at dose levels of 0 (vehicle), 1, 3 and 10 mg/kg/day once daily for 4 weeks, and 30 mg/kg/day once daily for 3 weeks (*N* = 5). The skin colour on the face was measured 3 times by Colorimeter (Konica Minolta) and the mean of the 3 measurement values was regarded as the L* value. The ΔL* represents the differences of individual L* values between each time point and pre‐test.

### 2.9 Pharmacokinetic studies

Blood was collected after single oral dosing of MT‐7117 was administered to 7‐week‐old male Ay/a mice (*N* = 4) and after 4‐weeks’ repeated oral dosing of monkeys in the facial skin colour study (*N* = 5). Plasma concentrations of MT‐7117 were determined using a high‐performance liquid chromatography/tandem mass spectrometry system (AB Sciex, API 4000 or 4000 QTRAP).

### 2.10 Statistical analysis

To evaluate the effects of the test substances, Dunnett's multiple comparison test, Williams' multiple comparison test or Fisher's exact test were performed to compare the treated groups with the controls. To estimate the EC_50_ values and their 95% confidence intervals, nonlinear regression analysis was performed. The geometric mean and 95% confidence interval of the EC_50_ value were determined from the data obtained in the 3 experiments. All analyses were done using the SAS system, and tests were two‐tailed with a significance level of <0.05 or one‐tailed with a significance level of <0.025.

## 3 RESULTS

### 3.1 Binding affinity and agonistic activity of MT‐7117 for MCRs

The binding affinity of MT‐7117 for a series of human MCRs was evaluated using a competitive binding assay with [^125^I] NDP‐αMSH. MT‐7117 showed the highest affinity for hMC1R with a Ki value of 2.26 nmol/l (Table 1). The Ki values of MT‐7117 for hMC3R, hMC4R and hMC5R were 1420, 32.9 and 486 nmol/l, respectively, demonstrating selectivity of MT‐7117 for MC1R (Table 1). In contrast, while NDP‐αMSH showed the highest affinity for MC1R with a Ki value of 0.028 nmol/l, its binding affinity for hMC3R, hMC4R and hMC5R were 0.17, 0.20 and 0.21 nmol/l, respectively, indicating its affinity across MCRs was also in the sub‐nanomolar range (Table 1).

**TABLE 1:** Binding activity of MT‐7117 for human recombinant melanocortin receptors

Human recombinant receptor	Ki value for receptor binding (nmol/l)	
	MT‐7117	NDP‐αMSH
MC1R	2.26	0.028
MC3R	1420	0.17
MC4R	32.9	0.20
MC5R	486	0.21

**FIGURE 2:**
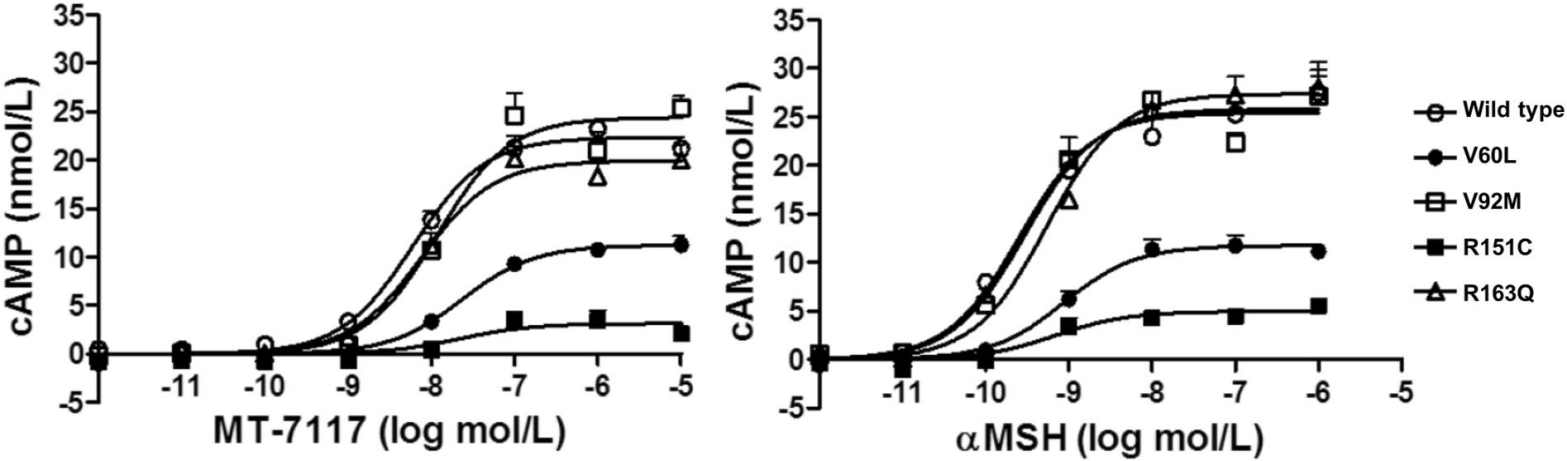
Effects of MT‐7117 on intracellular cAMP production in HEK293 cells transfected with stable expression of human MC1R variants. αMSH was used as the reference. Data are mean ± SEM of triplicate wells. Results are representatives of three independent experiments with similar findings

Agonistic activities of MT‐7117 and αMSH were evaluated on the basis of their ability to increase intracellular cAMP in HEK293 cells that stably express hMC1R with wild‐type or representative single‐nucleotide polymorphisms. Both test substances increased cAMP in a concentration‐dependent manner in cells with variants of MC1R and wild type, although the response of V60L and R151C mutants were weaker than that of the wild type MC1R (Figure 2). The Emax values of MT‐7117 reached similar levels to those of αMSH, indicating that the agonistic efficacy of MT‐7117 is comparable with that of αMSH (Figure 2).

To reveal species difference in agonistic activity, the EC_50_ values of MT‐7117, αMSH and NDP‐αMSH were determined for MC1Rs of several species. Agonistic activity for hMC4R was also evaluated since this receptor showed certain affinity to our compound (Table 1). MT‐7117 showed agonistic activity for hMC1R, cmMC1R and mMC1R, with EC_50_ values of 8.16, 3.91 and 1.14 nmol/l, respectively (Table 2). In contrast, the EC_50_ value of MT‐7117 for hMC4R was 79.6 nmol/l (Table 2) and the EC_50_ value for hMC2R was >10 000 nmol/l (data not shown). The agonistic activity of MT‐7117 for rat MC1R was also determined, with an EC_50_ value of 0.251 nmol/l (data not shown).

**TABLE 2:** Comparison of agonistic activity of MT‐7117 with αMSH and NDP‐αMSH for stably expressing melanocortin receptors on HEK293

	EC_50_ (nmol/l)			
	hMC1R	cmMC1R	mMC1R	hMC4R
αMSH	0.203	9.84	3.17	45.1
	(0.166–0.250)	(3.10–31.2)	(2.01–5.01)	(10.8–188)
NDP‐αMSH	0.0564	0.205	0.0524	0.764
	(0.0426–0.0748)	(0.0670–0.628)	(0.0274–0.100)	(0.429–1.36)
MT‐7117	8.16	3.91	1.14	79.6
	(6.74–9.88)	(1.77–8.63)	(0.259–5.06)	(44.3–143)

*Note*: The EC_50_ values were expressed as the geometric mean (95% confidence interval) of three independent experiments.

Abbreviations: cmMC1R, cynomolgus monkey MC1R; hMC1R, human MC1R; hMC4R, human MC4R; mMC1R, mouse MC1R.

### 3.2 Melanin production in B16F1 cells

**FIGURE 3:**
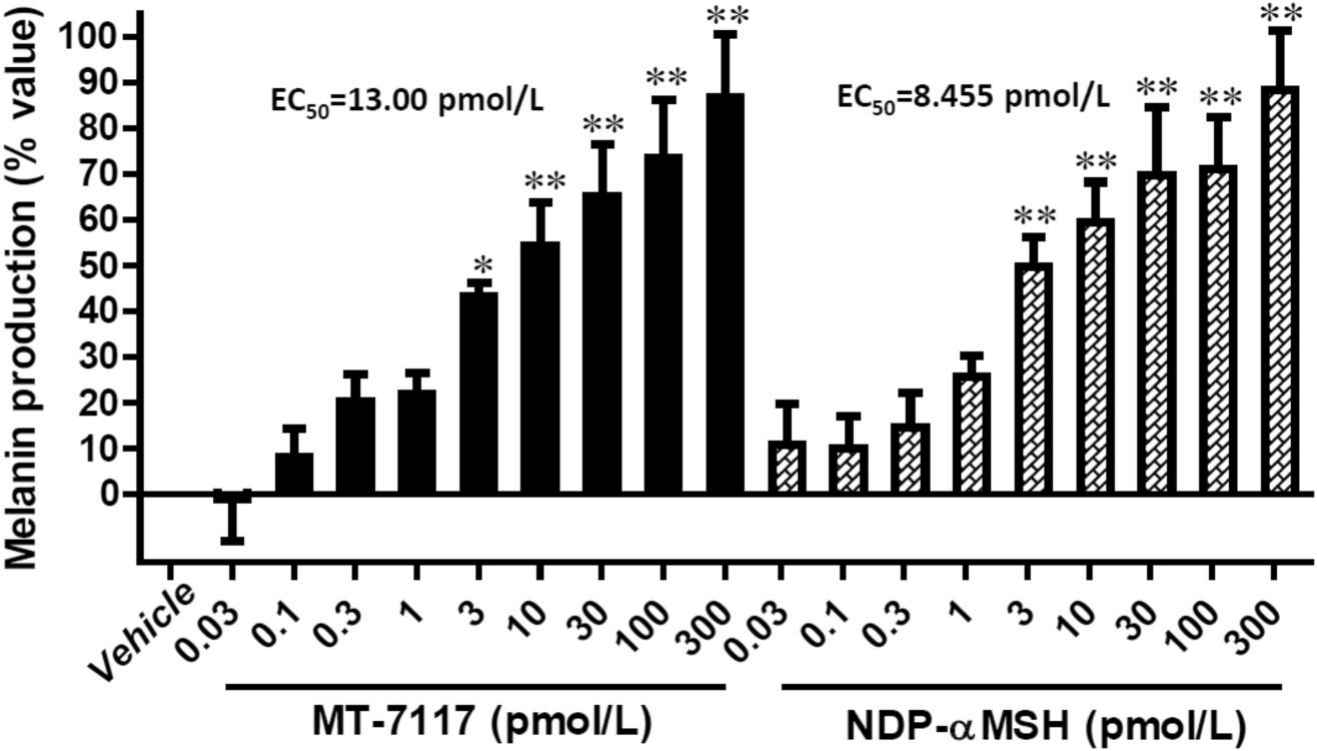
Effects of MT‐7117 on melanin production in B16F1 cells. Cells were treated with the indicated concentrations of test substances for 3 days. The % value is the percentage of melanin production relative to the maximum melanin production induced by NDP‐αMSH calculated by nonlinear regression analysis. Data are mean ± SEM of three independent experiments. *p < 0.05 and **p < 0.01 versus vehicle by Dunnett's multiple comparison test

To evaluate the ability of MT‐7117 to induce eumelanin production, B16F1 cells were incubated for 3 days with MT‐7117 or NDP‐α‐MSH, and the melanin concentration in the supernatant was determined. Both MT‐7117 and NDP‐αMSH at concentrations of ≥3 pmol/l significantly increased eumelanin production in a concentration‐dependent manner, with EC_50_ values (95% confidence interval) of 13.00 (0.2865–590.2) and 8.455 (0.4249–168.3) pmol/l, respectively (Figure 3).

### 3.3 Coat colour darkening of Ay/a mice

**FIGURE 4:**
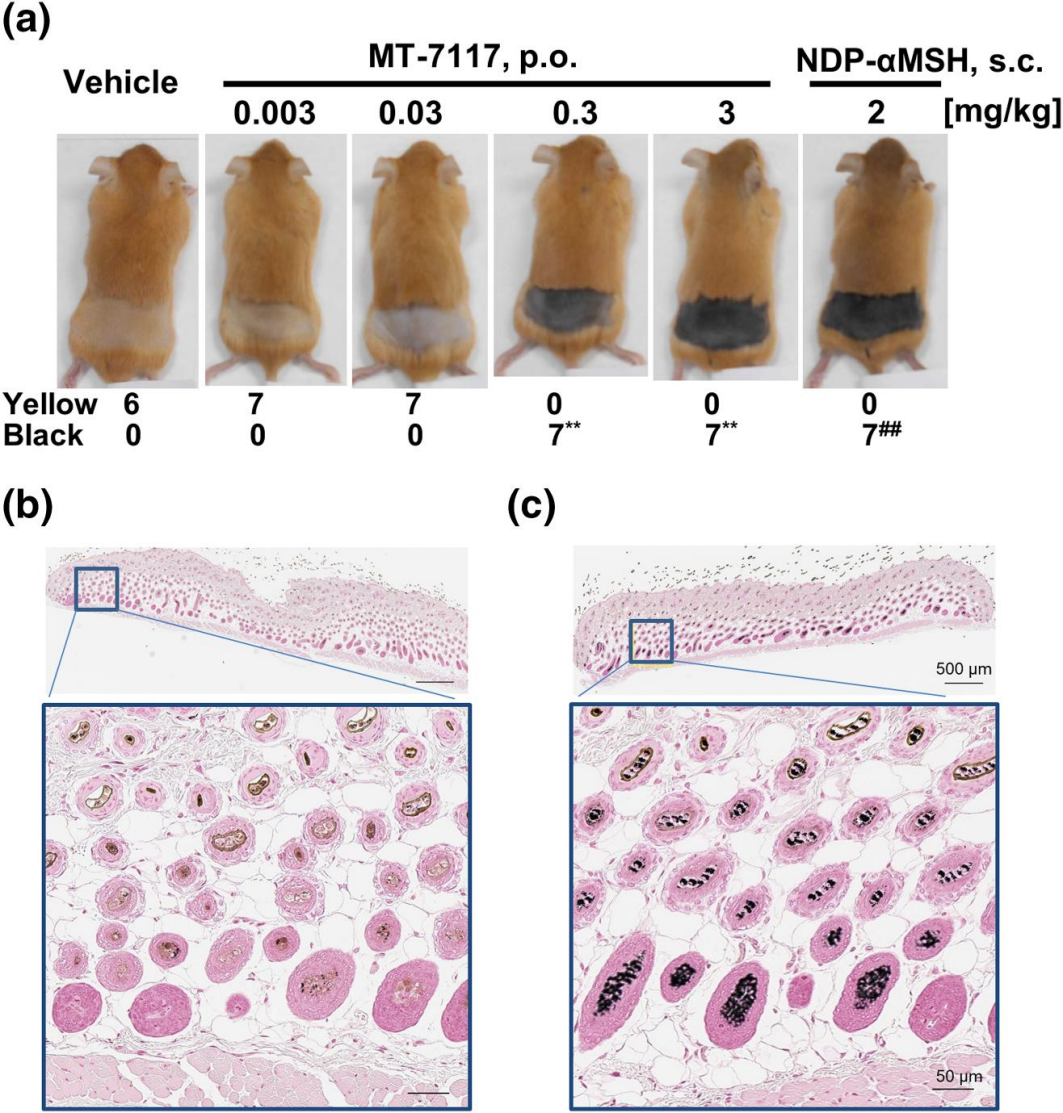
Effects of MT‐7117 on the coat colour darkening of Ay/a mice. MT‐7117 and NDP‐αMSH were administered for 6 days and, then, the coat in the dorsal area was shaved and the colour of the newly grown coat was assessed as yellow or black. ^##^ *p* < 0.01 versus vehicle by Fisher's exact test, **p < 0.01 versus vehicle by Fisher's exact test with multiplicity adjusted using fixed sequence procedure. (a) Representative image of each group on day 6 (above) and the number of mice with the emergent coat colour determined as yellow or black (below). (b) Representative image of Fontana‐Masson staining of dorsal skin in the vehicle‐treated group on day 6. The pigment in the hair root was brownish yellow. (c) Similar image to (b) from the MT‐7117 group treated at 3 mg/kg. The pigment in the hair root was black

MC1R agonists, such as αMSH, increase melanogenesis in the hair root and change the colour of the emergent coat from a normal yellow into dark brown in Ay/a mice.^16^, ^17^ The effects of MT‐7117 and NDP‐αMSH on coat colour were examined in this study. MT‐7117 was administered orally or NDP‐αMSH was subcutaneously administered for 6 consecutive days. The colour of the newly grown coat was assessed as yellow or black. The coat colour of all mice was determined as black in the 0.3 and 3 mg/kg MT‐7117‐ and 2 mg/kg NDP‐αMSH‐treated groups with statistical significance, indicating that MT‐7117 induced coat colour darkening in Ay/a mice (Figure 4a). To verify such an effect, histological examination of the hair root in the dorsal skin was performed and melanin was stained by the Fontana‐Masson procedure. The pigmentation in hair roots of representative animals in the vehicle group was brownish yellow (Figure 4b), in contrast, that in 3 mg/kg MT‐7117‐treated group was black (Figure 4c). These results suggest that MT‐7117 elicited synthesis of eumelanin rather than pheomelanin in hair roots in Ay/a mice.

### 3.4 Pharmacodynamic markers of melanisation

**FIGURE 5:**
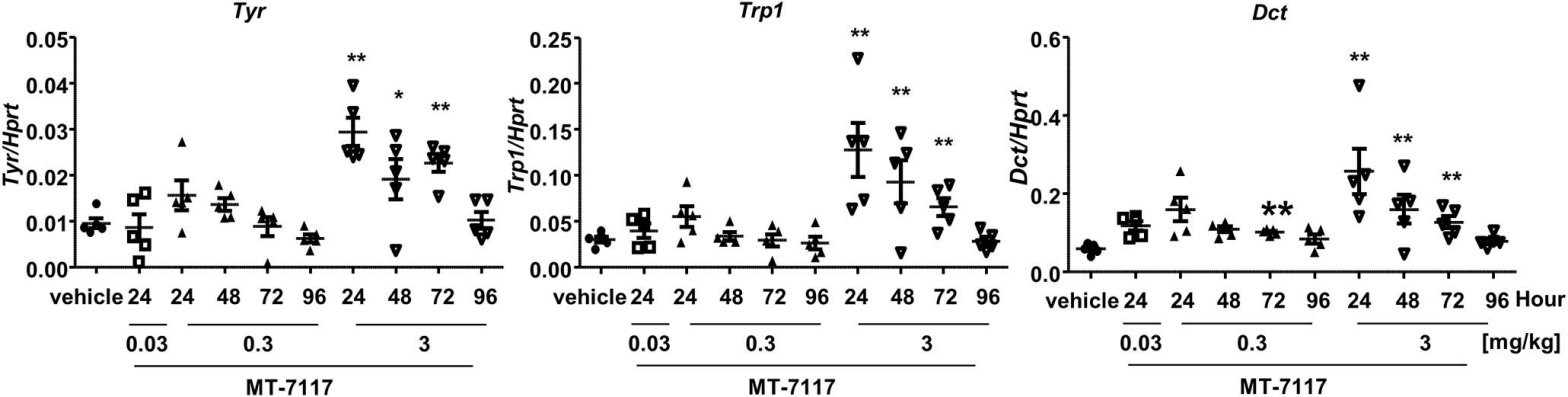
Effects of MT‐7117 on expression of genes related to melanogenesis in the pinnae from Ay/a mice. MT‐7117 was single administered orally to mice at 0.03, 0.3 and 3 mg/kg; thereafter, the pinnae were dissected at the time points indicated. The time point for the vehicle‐treated group was 72 h. Expression of *Tyr*, *Trp1* and *Dct* genes was determined relative to hypoxanthine phosphoribosyltransferase 1 (*Hprt*). Data are mean ± SEM (*N* = 5). *p < 0.025 and **p < 0.005 versus vehicle by Williams' multiple comparison test

To better understand how MT‐7117 influences melanisation in mice, the time course of gene expression related to eumelanin synthesis was examined in pinnae after single administration of MT‐7117. *Tyr*, *Trp1* and *Dct* are mainly involved in eumelanin biosynthesis.^1^ Expression of all three genes were significantly upregulated at 24, 48 and 72 h in the 3 mg/kg MT‐7117‐treated group, while a tendency towards gene upregulation was detected at 24 h after 0.3 mg/kg MT‐7117 (Figure 5).

### 3.5 Skin pigmentation in cynomolgus monkeys

**FIGURE 6:**
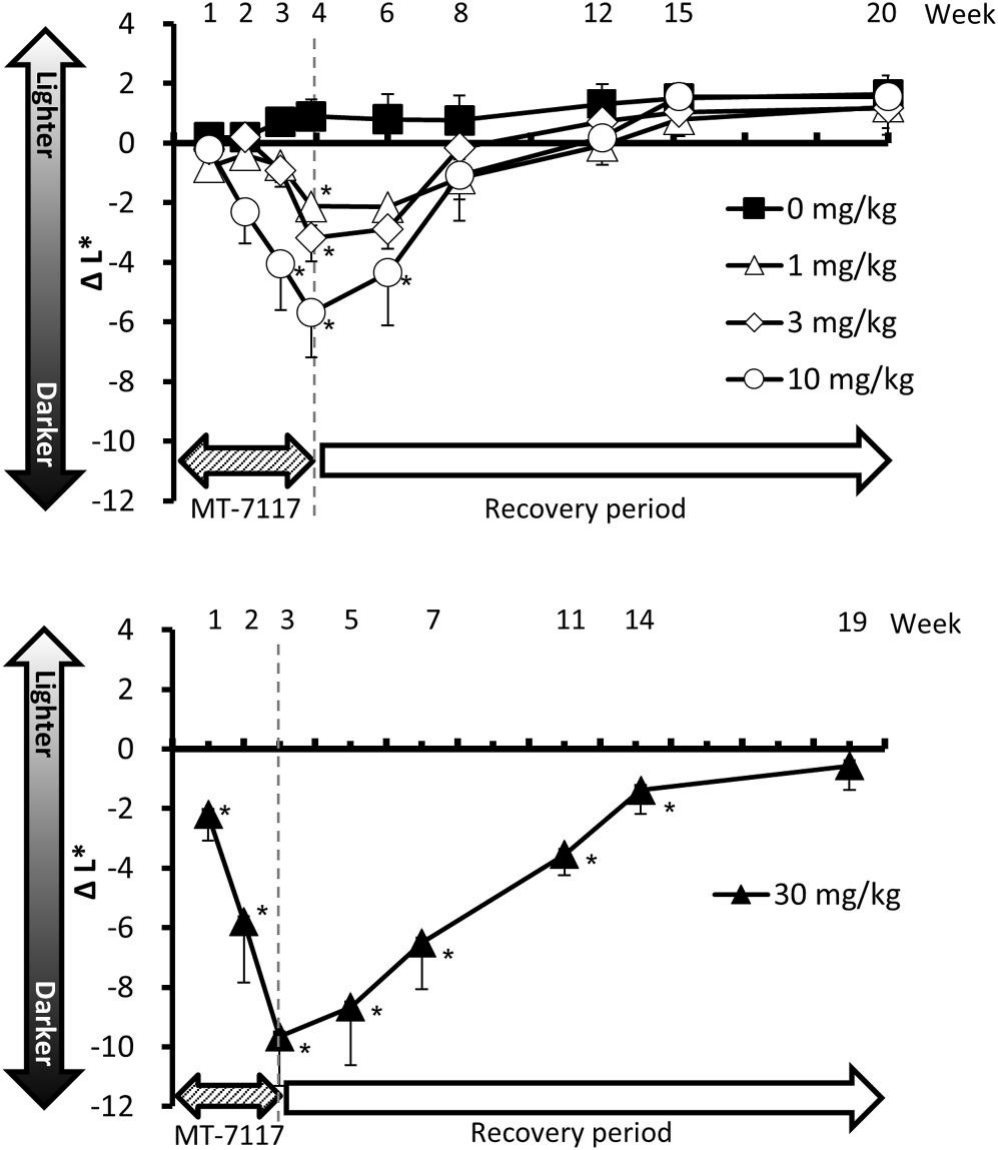
Effect of MT‐7117 on skin pigmentation in cynomolgus monkeys. MT‐7117 was administered orally to monkeys at 0 (vehicle), 1, 3 and 10 mg/kg for 4 weeks and 30 mg/kg for 3 weeks. Thereafter, the administration was stopped. Changes in ΔL* values, the differences of individual L* values between each time point and pre‐test, are shown. Data are mean ± SEM (*N* = 5). *p < 0.025 versus vehicle by Williams' multiple comparison test

The induction and reversibility of pigmentation of facial skin of cynomolgus monkeys induced by MT‐7117 was measured by a colorimeter. During the dosing period, statistically significant decrease of ΔL* values as compared with the vehicle group were noted in the 30 mg/kg‐treated group after 1 week, in the 10 mg/kg‐treated group from after 3 weeks and in the 1 and 3 mg/kg‐treated groups after 4 weeks (Figure 6). Pigmentation diminished 4 weeks after cessation of treatment in the 1, 3 and 10 mg/kg groups and 16 weeks after cessation in the 30 mg/kg group (Figure 6). These results indicate that pigmentation induced by MT‐7117 was dose proportional and its minimum effective dose was 1 mg/kg, and it was reversed after cessation of administration.

### 3.6 Pharmacokinetic studies

Plasma concentrations of MT‐7117 were determined after a single dose in Ay/a mice and after 4 weeks’ repeated dosing in cynomolgus monkeys. Pharmacokinetic parameters are summarised in Table 3. Plasma levels of MT‐7117 in mice increased in a dose‐proportional manner, while the rise in levels in monkeys was greater than a dose‐proportional increase (Table 3).

**TABLE 3:** Pharmacokinetic parameters of MT‐7117 after oral administration to Ay/a mice and cynomolgus monkeys

Species/strain	Dose (mg/kg)	Day	Sex	*C*_max_ (ng/ml)	*t*_max_ (h)	AUC_0–8 h_ (ng·h/ml)	AUC_0–24 h_ (ng·h/ml)
Mouse/Ay/a	0.03	1	M	0.668	0.5	2.62	
	0.3	1	M	5.62	2	31.8	
Monkey/cynomolgus	1	28	F	53.0	2.6		428
	3	28	F	401	1.0		2510
	10	28	F	1510	1.8		14 100

*Note*: Pharmacokinetic parameters in mice are expressed as the mean of four animals. Pharmacokinetic parameters in monkeys are expressed as the mean of five animals.

Abbreviations: AUC_0–8 h_, area under the plasma concentration–time curve from zero up to 8 h after dosing; AUC_0–24 h_, area under the plasma concentration–time curve from zero up to 24 h after dosing; *C*_max_, maximum plasma concentration after administration; F, female; M, male; *t*_max_, time to reach maximum plasma concentration after administration.

## 4 DISCUSSION

We have described the pharmacological profile of a novel oral MC1R agonist, MT‐7117, in experimental models of melanin production. MT‐7117 behaved as a full agonist for hMC1R in the recombinant cell assays as demonstrated by the observation that Emax of MT‐7117 reached the same level as that of the endogenous agonist αMSH (Figure 2). Interestingly, MT‐7117 induced melanin production in B16F1 cells with an EC_50_ value close to that of NDP‐αMSH (Figure 3), while the EC_50_ value of cAMP production by MT‐7117 was approximately 20‐fold less potent than that seen with NDP‐αMSH in mMC1R‐transfected cells (Table 2). For clarification, we examined cAMP production by MT‐7117 or NDP‐αMSH in B16F1 cells and found that the EC_50_ value of MT‐7117 was similarly 19‐fold less potent than that of NDP‐αMSH (Figure S1), which was consistent with the result from mMC1R‐transfected cells. We speculate that distinct intracellular signalling pathways such as Erk1/2 and Akt phosphorylation other than cAMP production reported in the literature^12^ are involved in MT‐7117‐induced melanisation in B16F1 cells. Further analysis will be needed to clarify the precise mechanism of agonistic signalling of MT‐7117 to learn how it is different from peptide agonists.

The minimum effective doses of MT‐7117 were 0.3 and 1 mg/kg for the darkening of coat colour of mice and skin pigmentation of monkeys, respectively (Figures 4 and 6). When we measured the serum levels of MT‐7117 after single oral dosing in mice, Cmax reached 5.6 ng/ml (= 8.3 nmol/l) at 0.3 mg/kg (Table 3). In regards to the plasma levels of MT‐7117 after repeated oral dosing to monkeys, Cmax reached 53 ng/ml (= 78 nmol/l) at 1 mg/kg (Table 3). These results indicate that the level of systemic exposure of MT‐7117 reasonably exceeded the EC_50_ values of MC1R agonistic activity, which represent the target level necessary to exhibit efficacy in the body. Moreover, the extent of darkness of skin pigmentation of monkeys became greater and quicker dose‐proportionally up to 30 mg/kg of MT‐7117 without reaching saturation (Figure 6). Once the administration was stopped, reversal of skin pigmentation was observed over the course of 19 weeks with the degree of reversal dependent on the dosage. Skin turnover period is reported to take 4–5 weeks.^18^ Thus, the degree of pigmentation induced by MT‐7117 will be influenced by several factors such as dosage, frequency, duration and kinetics of epidermal turnover, suggesting that optimal administration for therapeutic usage should be explored in clinical studies in order to maintain sufficient levels of melanin over time to prevent phototoxic reactions caused by sunlight.

There is concern about the risk of melanoma by overstimulation of pigmentation. In nonclinical, αMSH showed neither increase cell proliferation nor invasion of mouse melanoma in vitro.^19^ We found that MT‐7117 showed no effect on cell proliferation in 5 human melanoma cell lines in vitro (data not shown). Moreover, there was no histopathological change for melanocytes in face, melanocyte abundant region in chronic toxicity study in non‐rodent species (data not shown). Hence, there is no evidence of melanoma risk for MT‐7117 in nonclinical, although further studies are needed to warrant the safety. Epidemiological evidence indicates that mutations in MC1R are genetic risk factors for melanoma in humans that are thought to act by loss of function.^20^, ^21^ The αMSH/MC1R agonism has a role of prevention of skin cancer including melanoma by up regulating two important photo‐protective mechanisms, DNA repair and pigmentation.^10^, ^22^ Therefore, impaired function rather than activation of MC1R may increase the vulnerability of melanocytes to UV‐induced transformation to malignant melanoma.

Apart from pigmentation, various biological effects of endogenous ligand αMSH have been reported in nonclinical settings, such as anti‐inflammatory effects,^2^, ^23^ anti‐fibrotic effects,^24^, ^25^ wound healing effects,^26^ and enhancement of nucleotide excision repair.^10^, ^22^ These effects suggest that αMSH plays a broad protective role in homoeostatic maintenance in the body. Future studies will determine if MT‐7117 can elicit similar biological effects.

MT‐7117 showed high selectivity for MC1R, and to some extent agonistic activity for MC4R (Table 2). MT‐7117 was less likely to be distributed into the brain, which is the predominant tissue of MC4R expression, in a tissue distribution study in rats (data not shown). Hence, based on specificity and distribution, we expect MT‐7117 to act as a selective MC1R agonist in the body. In contrast, NDP‐αMSH binds predominantly to MC1R but still behaves as a pan‐MCR agonist, which may cause unfavourable side effects such as nausea and this could be explained by its binding to MC3R.^14^

Although NDP‐αMSH is currently used to treat adult patients with EPP, there remains a high medical need for new effective, safe and more conveniently administered treatments for EPP and XLP, especially for pediatric/adolescent patients who have had no options to date. The oral route of administration of MT‐7117 is a salient feature that provides ease of administration and results in consistent therapeutic levels of the compound. In conclusion, we have designed MT‐7117, a novel and selective oral MC1R agonist. MT‐7117 is currently being assessed in a phase 3 trial (NCT04402489) as a potential new therapeutic option to permit an increase in pain‐free light exposure for adults and adolescents with a history of phototoxic reactions from EPP or XLP.

## CONFLICT OF INTEREST

All authors are employees of Mitsubishi Tanabe Pharma Corporation.

## AUTHOR CONTRIBUTIONS

**T. Suzuki:** Conceptualization; Data curation; Formal analysis; Investigation; Methodology; Project administration; Supervision; Validation; Visualization; Writing – original draft; Writing – review & editing. **Y. Kawano:** Conceptualization; Data curation; Investigation; Methodology; Project administration; Software; Validation; Writing – original draft; Writing – review & editing. **A. Matsumoto:** Data curation; Formal analysis; Investigation; Methodology; Software; Validation; Visualization; Writing – original draft; Writing – review & editing. **M. Kondo:** Conceptualization; Data curation; Formal analysis; Investigation; Methodology; Project administration; Software; Validation; Visualization; Writing – original draft; Writing – review & editing. **K. Funayama:** Investigation; Methodology; Software; Validation. **S. Tanemura:** Investigation; Methodology; Software; Validation. **M. Miyashiro:** Conceptualization; Investigation; Methodology; Project administration; Resources; Software; Supervision; Validation; Writing – review & editing. **A. Nishi:** Conceptualization; Investigation; Methodology; Project administration; Resources; Software; Supervision; Validation; Writing – review & editing. **K.** **Yamada:** Investigation; Methodology; Software; Validation; Writing – review & editing. **M. Tsuda:** Investigation; Methodology; Software; Validation; Writing – original draft; Writing – review & editing. **A. Sato:** Investigation; Methodology; Validation. **K. Morokuma:** Investigation; Methodology; Validation. **Y. Yamamoto:** Investigation; Methodology; Project administration; Resources; Supervision; Validation.

## Supporting information

Figure S1

## Data Availability

Research data are not shared.
