# Supplementary figures and images for: Melanogenic effect of dersimelagon (MT‐7117), a novel oral melanocortin 1 receptor agonist
Source: Skin Health Dis. 2021 Nov 29;2(1):e78. doi: 10.1002/ski2.78 (PMC9060023; doi:10.1002/ski2.78)

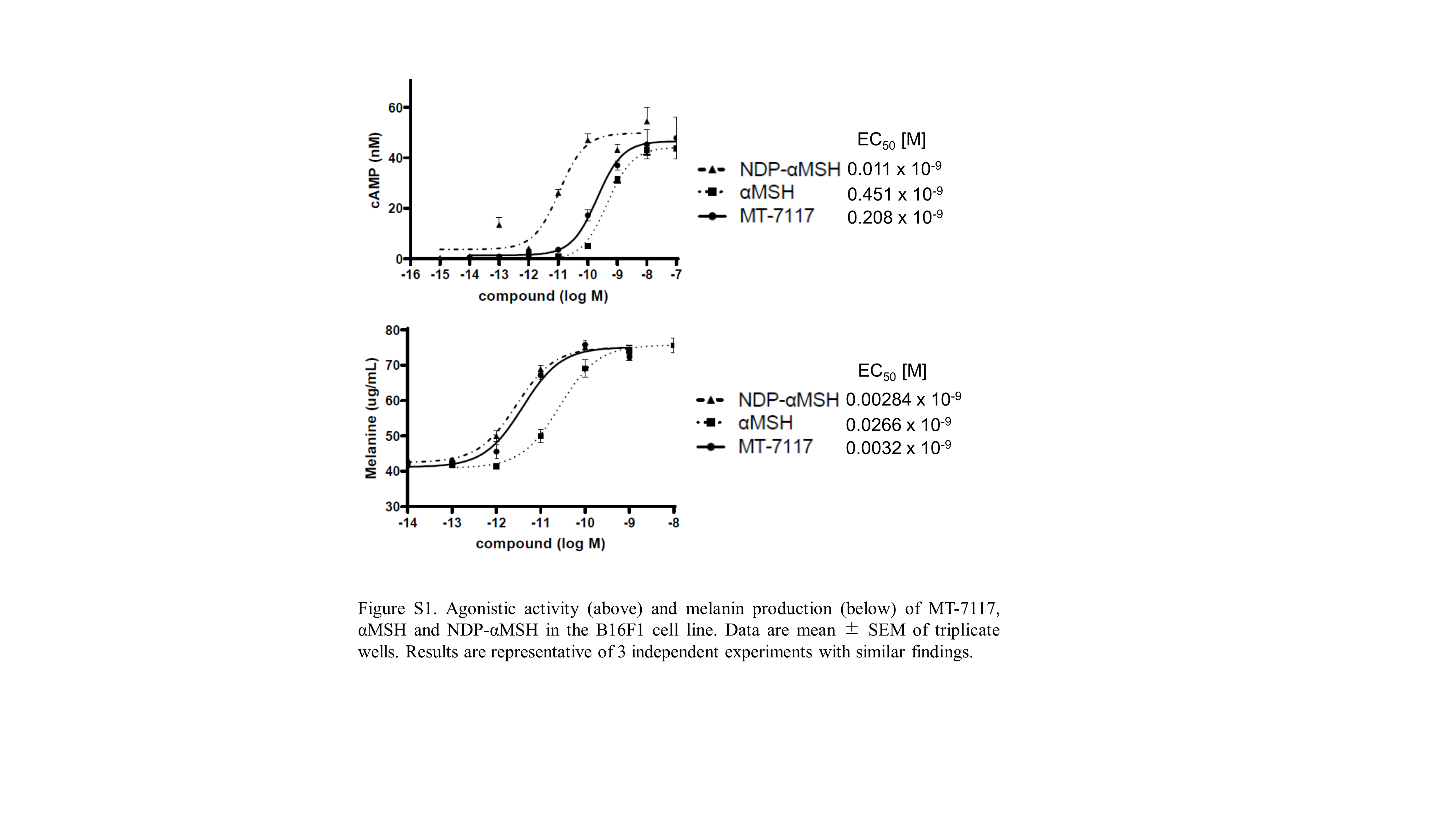

Supplement: Supplementary file 1 — Figure S1 [file SKI2-2-e78-s001.tif]
